# *TMEM164* mRNA in Adult Glioblastoma: Multicohort Overexpression and *RIPK1* Co-Expression Without Prognostic Replication

**DOI:** 10.3390/brainsci16101049

**Published:** 2026-09-29

**Authors:** Flavio Donnini, Cristiana Bellan, Sergio Antonio Tripodi, Lucia Mundo, Paolo Tini

**Affiliations:** 1 Unit of Radiation Oncology, Department of Medicine, Surgery and Neurosciences, University of Siena, Azienda Ospedaliero-Universitaria Senese, 53100 Siena, Italy; 2 Unit of Pathological Anatomy, Department of Medical Biotechnologies, University of Siena, Azienda Ospedaliero-Universitaria Senese, 53100 Siena, Italylucia.mundo.ml@gmail.com (L.M.)

**Keywords:** glioblastoma, *TMEM164*, *RIPK1*, necroptosis, radioresistance, prognostic biomarker, meta-analysis, secondary analysis

## Abstract

**Highlights:**

**What are the main findings?**

- *TMEM164* mRNA occupied a higher within-sample rank in glioblastoma than in non-tumour brains in two independent comparisons, the two CGGA cohorts sharing a single control arm, and is co-expressed with *RIPK1* in all three cohorts; in TCGA, this correlation ranks 43rd of 19,985 genes and is unchanged by adjustment for immune, myeloid, stromal and endothelial content.
- Baseline *TMEM164* mRNA carries no reproducible prognostic information: the cohort estimates diverge in direction, and a hazard ratio of 1.8 can be manufactured in the same data by optimal-cutpoint selection and disappears once that selection is corrected for.

**What are the implications of the main findings?**

- A dichotomised single-database Kaplan–Meier claim is not adequate evidence that a transcript is prognostic in glioblastoma, and the present data set the boundary on how large any *TMEM164* effect could still be.
- Because the proposed radioresistance mechanism is post-translational and TMEM164 protein is not quantified in any public glioblastoma proteome, evaluation of *TMEM164* as a target requires protein- and activity-level assays on treatment-related tissue sampling.

**Abstract:**

**Background/Objectives:** Experimental work has implicated TMEM164 in glioblastoma radioresistance through post-translational inhibition of FASN and suppression of radiation-induced necroptosis, supported clinically by a dichotomised single-database survival analysis. We tested whether the previously reported transcript-level clinical association of *TMEM164* is reproducible across independent human glioblastoma cohorts and characterised its relationship with tumour status, necroptosis-related transcripts and recurrence. **Methods:** Secondary analysis of four public resources: TCGA (*n* = 153), CGGA-693 (*n* = 104), CGGA-325 (*n* = 73), and 104 matched primary-recurrence pairs from GLASS. Overall survival was modelled by continuous Cox regression, pooled by Hartung–Knapp random-effects meta-analysis. **Results:** *TMEM164* occupied a higher within-sample percentile in tumour than in non-tumour brains in all three cohorts (Cliff’s δ 0.68–0.85; all *p* ≤ 1.3 × 10^−3^), was co-expressed with *RIPK1* (ρ 0.25–0.44; all q < 0.05), ranking 43rd of 19,985 genes, and was unchanged by adjustment for tumour composition. No association with overall survival replicated across cohorts, in the pooled analysis (HR 1.11, 95% CI 0.77–1.61; I^2^ = 49.9%), among irradiated patients or in confirmed *IDH*-wildtype cases; isolated nominally significant positive estimates arose in exploratory strata and were not reproduced elsewhere. With 277 events, the pooled interval excludes hazard ratios above approximately 1.6 per standard deviation, while smaller effects remain compatible. An optimal cutpoint generated HR 1.83 (*p* = 0.004) that did not survive correction for selection. TMEM164 protein was quantified in 0 of 99 CPTAC glioblastomas. **Conclusions:** Baseline bulk-tumour *TMEM164* mRNA carries no reproducible prognostic information in adult glioblastoma. The proposed mechanism is post-translational and must be tested at the protein and activity level, on treatment-related tissue sampling.

## 1. Introduction

Glioblastoma, *IDH*-wildtype, is the most aggressive primary malignant brain tumour in adults and remains associated with poor survival despite maximal safe resection followed by radiotherapy and temozolomide [1,2,3]. Radioresistance is sustained by efficient DNA-damage responses, stem-like cellular states, and microenvironmental adaptation, and almost all tumours ultimately recur [3,4]. Identifying mechanisms that are biologically actionable and measurable in patients is therefore a major translational priority.

Transmembrane protein 164 (TMEM164) has emerged as a regulator of lipid remodelling and regulated cell death [5]. In cancer models, TMEM164 promotes autophagy-dependent ferroptosis by selectively mediating ATG5-dependent autophagosome formation, and high TMEM164 expression is associated with improved survival in pancreatic cancer [6]. Structural studies subsequently identified TMEM164 as a seven-transmembrane acyltransferase that remodels polyunsaturated ether phospholipids, characterised it as anti-ferroptotic, and identified an experimental S-enantiomer of montelukast as a candidate inhibitor [7]. The direction of effect attributed to TMEM164 therefore differs between these two reports, pro-ferroptotic in the first and anti-ferroptotic in the second, as well as between tumour types. Both nonetheless provide a defined enzymatic framework for therapeutic investigation.

In glioblastoma cell lines, Jiang et al. reported that fractionated irradiation induced TMEM164 and that TMEM164 enhanced radioresistance by suppressing radiation-induced necroptosis [8]. Every step of the proposed mechanism operates downstream of transcription. TMEM164 was reported to interact with FASN and to inhibit its enzymatic activity, producing NADPH accumulation, neutralisation of radiation-induced reactive oxygen species, and reduced phosphorylation of the necroptosis effectors RIPK1, RIPK3 and MLKL, which were measured as phosphoproteins rather than as transcripts [8]. The supporting clinical evidence, by contrast, rested on a dichotomised single-database Kaplan–Meier comparison with an unspecified threshold, a design known to generate cutpoint-dependent associations [9]. Necroptosis itself is context-dependent in cancer and can support either tumour-cell death or inflammation-mediated progression [10,11].

This asymmetry, between a post-translational mechanism and a transcript-level clinical claim, defines the question addressed here. We conducted a secondary analysis of four independent public transcriptomic resources to determine whether bulk-tumour *TMEM164* mRNA is elevated relative to non-tumour brains, co-expressed with *RIPK1*, *RIPK3*, *MLKL* or *FASN*, associated with overall survival under continuous cutpoint-free models, altered between matched primary and recurrent tumours, or associated with survival specifically among irradiated patients. We further asked whether an optimal-cutpoint analysis can reproduce a positive association in these same data, and whether the protein level at which the mechanism operates is accessible in existing public proteomic resources. The aim is not to test the experimental mechanism, which transcriptomic data cannot address, but to establish what baseline bulk-tumour transcription can and cannot contribute to the evaluation of *TMEM164* in patients. Accordingly, failure of *TMEM164* mRNA to predict outcome should not be interpreted as evidence against a functional role of *TMEM164* protein in radioresistance; it would instead indicate that baseline transcript abundance is an inadequate clinical surrogate for that mechanism.

## 2. Materials and Methods

### 2.1. Study Design and Data Sources

This was a secondary analysis of four publicly available transcriptomic resources; no new patient data were generated. TCGA-GBM RNA-sequencing and curated clinical data were retrieved through the UCSC Xena platform [12], which hosts data generated by The Cancer Genome Atlas [13]. The Chinese Glioma Genome Atlas (CGGA) mRNAseq_693 and mRNAseq_325 datasets, together with the dedicated non-glioma control dataset, were downloaded from the CGGA portal [14]. The Glioma Longitudinal Analysis Consortium (GLASS) dataset [15] was accessed through cBioPortal [16,17]. Somatic mutation data and CPTAC proteomic data were likewise obtained through cBioPortal.

### 2.2. Cohort Definition and Data Curation

TCGA cases were restricted to primary, treatment-naive histologic glioblastoma with available *TMEM164* expression and overall-survival data. The cohort was audited at sample level. The expression matrix contained 172 samples: 154 primary tumours (sample type code 01), 13 recurrent tumours (code 02) and 5 non-tumour brain samples (code 11). All 13 recurrent samples were excluded—6 of them belonged to patients who were also represented by their primary sample and carried identical survival time and vital status, so including them would have entered those patients into the survival model twice with identical follow-up, while the remaining 7 were patients represented only by a recurrent specimen. One further primary sample (TCGA-28-2510-01) was excluded because it is absent from the curated survival matrix. The resulting cohort comprised 153 patients with 121 deaths, and the complete case flow is given in Supplementary Table S1. Treatment-naive status is documented rather than inferred from the sample type code: all 153 cases are annotated as primary tumour with no history of neoadjuvant treatment, although 5 carry a recorded history of prior glioma. Five non-tumour brain samples from the same expression matrix (TCGA-06-0675-11, TCGA-06-0678-11, TCGA-06-0680-11, TCGA-06-0681-11, TCGA-06-AABW-11) served as a within-platform reference.

Molecular *IDH* status was determined for the TCGA cohort from PanCancer Atlas somatic mutation data accessed through cBioPortal, using Entrez Gene identifiers 3417 (*IDH1*) and 3418 (*IDH2*). Any non-synonymous variant in either gene was counted as *IDH*-mutant, without restriction to canonical codons; this conservative definition avoids dependence on hotspot annotation. Mutation data were available for 151 of 153 cases (98.7%), identifying 8 *IDH*-mutant and 143 confirmed *IDH*-wildtype tumours, with 2 cases not profiled and retained as a separate category. The resulting classification is consistent with the curated molecular characterisation of TCGA diffuse glioma [18].

CGGA cohorts were restricted to primary, molecularly *IDH*-wildtype, WHO grade IV tumours in patients aged 18 years or older. The adult age filter excluded one paediatric case from each cohort, yielding 104 patients with 92 deaths in CGGA-693 and 73 patients with 64 deaths in CGGA-325. Twenty non-glioma brain samples from the dedicated CGGA control dataset (Illumina HiSeq, STAR + RSEM pipeline) served as the reference for both CGGA cohorts.

For the longitudinal analysis, GLASS cases were restricted to molecularly *IDH*-wildtype tumours with RNA sequencing at initial diagnosis and first recurrence, yielding 104 matched pairs with complete tumour-purity annotation.

Cohort independence was verified directly. CGGA-693 and CGGA-325 shared no patient identifiers. GLASS contained 27 TCGA-format identifiers among 314 samples; 9 of the 104 pairs (8.7%) included at least one TCGA sample, and 6 of these patients were also present in the cross-sectional TCGA cohort. GLASS contained no CGGA identifiers. Because the same 20 CGGA controls served both CGGA tumour cohorts, the tumour-versus-non-tumour analysis comprises two independent comparisons (TCGA and CGGA), not three. Cohort composition and covariate availability are given in Supplementary Table S1.

### 2.3. Expression Processing

*TMEM164* expression was transformed within each dataset as log_2_(RSEM + 1) for TCGA, log_2_(FPKM + 1) for CGGA, and log_2_(TPM + 1) for GLASS. Because these units are not interchangeable, absolute expression values were never pooled across platforms; cross-cohort models used standardised (z-scored) expression, and tumour-versus-control comparisons were performed only within the same processing pipeline.

The CGGA control matrix was annotated over 55,523 genes, against 23,987 (CGGA-693) and 24,326 (CGGA-325) in the tumour matrices, with the tumour gene sets entirely contained within the control set. The additional genes contributed a negligible share of total signal (median fraction of library retained within the common gene space, 0.999). For absolute-scale comparisons, counts per million were therefore recomputed separately for each cohort over the corresponding common gene space and log_2_(x + 1)-transformed; control values were therefore derived separately for each comparison, although the resulting numerical difference between them is negligible (Supplementary Table S3).

### 2.4. Tumour Versus Non-Tumour Comparison

The concern with absolute-scale comparison is that library-size normalisation does not remove a global compositional shift between tumour and normal brain. Three constitutively expressed genes (*ACTB*, *GAPDH*, *TBP*) were therefore analysed alongside *TMEM164* as negative controls. The primary metric was the within-sample percentile rank of *TMEM164* among all genes measured in the same sample. This quantity is invariant to any monotone global rescaling and is therefore unaffected by platform-level or library-level shifts. Absolute-scale comparisons are reported as a secondary analysis. Because a transcript whose rank already lies in the upper tail of the distribution has limited room to move on a percentile scale, two further controls were applied to the percentile metric itself: the per-sample fraction of detected genes was compared between tumour and control samples, to verify that the denominator of the rank is comparable between groups; and the within-sample percentile rank was computed both for the three constitutively expressed transcripts above and for a panel of ten transcripts previously proposed as uniformly expressed human reference genes, listed individually in Supplementary Table S11. Group differences were tested with the two-sided Wilcoxon rank-sum test, evaluated from the normal approximation to the rank-sum distribution rather than from the exact permutation distribution—with only five TCGA control samples, this is the conservative choice, the exact test returning smaller *p* values than those reported. Effect sizes were quantified with Cliff’s δ and its 95% confidence interval [19].

### 2.5. Co-Expression Analyses

Spearman rank correlations quantified the association of *TMEM164* with *RIPK1*, *RIPK3*, *MLKL* and *FASN* in each cohort. Because of tied expression values, *p* values were computed from the asymptotic approximation; 95% confidence intervals were obtained by percentile bootstrap with 2000 resamples (seed 20260831). The resulting 12 tests (3 cohorts × 4 genes) were corrected using the Benjamini–Hochberg procedure [20]. No multiplicity correction was applied to any other analysis in this study; replication across independent cohorts was used instead, and this is stated explicitly wherever a nominal *p* value is reported.

Two analyses addressed whether *TMEM164*-*RIPK1* co-expression reflects cellular composition rather than a specific relationship. First, a transcriptome-wide null distribution was constructed in TCGA by computing the Spearman correlation of *TMEM164* with each of 19,985 analysable genes, and locating ρ(*TMEM164*, *RIPK1*) within it. This was performed in TCGA because that cohort yields the strongest observed correlation and therefore provides the condition least favourable to the null hypothesis. Second, partial Spearman correlations were computed adjusting for an immune/myeloid score (mean z-score of *PTPRC*, *AIF1*, *CD68*, *CSF1R*, *ITGAM*, *TYROBP*, *C1QA*, *C1QB*, *CD14*, *FCGR3A*), a stromal/endothelial score (*PECAM1*, *VWF*, *COL1A1*, *COL1A2*, *DCN*, *ACTA2*), and both jointly.

### 2.6. Survival Analysis and Model Diagnostics

Overall survival was modelled by Cox proportional-hazards regression with *TMEM164* entered as a continuous z-scored variable, so that hazard ratios express the effect of a 1-standard-deviation increase in expression. *TMEM164* was modelled as a continuous variable throughout and no data-driven dichotomisation was applied at any stage of the primary analysis; no written analysis plan was registered in advance of the study.

Three diagnostics were applied to every cohort-level model. The proportional-hazards assumption was tested using scaled Schoenfeld residuals, per term and globally. Departure from linearity was tested by refitting the model with a restricted cubic spline in *TMEM164* and comparing it with the linear model. Discrimination was quantified by Harrell’s concordance index, corrected for optimism by bootstrap with 2000 resamples (seed 20260831) [21]; both apparent and optimism-corrected values are reported, together with the 95% interval of the optimism estimate (Supplementary Table S4). The index is reported directionally rather than as a magnitude, so that values below 0.5 denote discrimination in the direction opposite to the modelled one. This matters in TCGA, where the point estimate for *TMEM164* lies below unity and the concordance is correspondingly below 0.5.

For TCGA, the primary multivariable model included *TMEM164*, age at diagnosis, and molecular *IDH* status (wildtype/mutant/not profiled), all available for the full cohort of 153 patients with 121 events. A secondary model substituted Karnofsky Performance Status for *IDH* status and was restricted to the 115 patients with complete KPS (89 events). Covariate completeness in every cohort is tabulated in Supplementary Table S1, and models were built from the covariates that were both available and substantially complete. As a design-stage reference, the minimum hazard ratio per standard deviation detectable with 80% power at a two-sided α of 0.05 was computed for each cohort and for the combined event count from the observed number of events. This quantity describes the sensitivity conferred by the sample sizes and is not used as the exclusion boundary for the observed synthesis, which is read from the confidence interval of the pooled estimate.

### 2.7. Meta-Analysis

Cohort-specific log hazard ratios, all expressed per standardised unit and therefore on a common scale, were combined by random-effects meta-analysis with restricted maximum-likelihood estimation of τ^2^ [22]. Because REML estimation of between-study variance is unstable with a small number of studies, the Hartung–Knapp–Sidik–Jonkman adjustment was used for primary inference [23], with the unadjusted REML model and a fixed-effect model reported as sensitivity analyses. Heterogeneity was summarised by Cochran’s Q and I^2^ [24], and leave-one-out analysis was performed to assess the influence of each cohort (Supplementary Table S5).

### 2.8. Cutpoint Analysis

To test whether a positive prognostic association can be produced in these same data by the analytic strategy used in the source study, an optimal cutpoint was identified in each cohort using maximally selected rank statistics. For each cohort, the optimal cutpoint, the corresponding hazard ratio, the uncorrected minimum *p* value, and the *p* value corrected for cutpoint selection are reported, using the approximation of Lausen and Schumacher [25] as recommended for prognostic-factor studies by Altman et al. [9], implemented in the maxstat package (version 0.7.26). The uncorrected *p* value is the log-rank test at the selected cutpoint, that is, the quantity an analysis reporting a data-driven dichotomisation would present.

### 2.9. Subgroup and Sensitivity Analyses

The analyses in this section were not planned in advance and were not corrected for multiplicity. They are exploratory, are reported in full including nominally significant results, and are interpreted accordingly.

Irradiated patients. Radiotherapy exposure was taken from the radiation_therapy field in the TCGA clinical matrix (124 treated, 21 untreated, 8 missing; 2 cases discordant with CDE_radiation_any were retained on the primary variable) and from the Radio_status field in both CGGA cohorts. Cohort-specific models and a meta-analysis were repeated in irradiated patients only. A *TMEM164* × radiotherapy interaction was tested by the likelihood-ratio test in each cohort. As a sensitivity analysis, the time origin was reset to 90 and to 60 days after diagnosis in the CGGA cohorts (Supplementary Table S6).

Molecular *IDH* status. The TCGA analysis was repeated in the 143 confirmed *IDH*-wildtype cases. 

*MGMT* promoter methylation. Separate models were fitted in methylated and unmethylated subgroups of both CGGA cohorts (Supplementary Table S10).

Extended CGGA-693 cohort. A cohort of primary, *IDH*-wildtype, adult cases with non-missing survival and WHO grade was assembled irrespective of grade (*n* = 164, 130 events). Models were fitted with and without adjustment for grade and age, within each grade stratum, and with a *TMEM164* × grade (WHO IV versus II-III) interaction tested by the likelihood-ratio test. The correlation between *TMEM164* and grade was assessed by Spearman correlation and by the Kruskal–Wallis test. The clinical annotation of CGGA-693 comprises histology, grade, age, sex, radiotherapy and chemotherapy status, *IDH* mutation, 1p/19q codeletion and *MGMT* promoter methylation; none of the three molecular criteria that define *IDH*-wildtype glioblastoma under the 2021 WHO classification (*TERT* promoter mutation, *EGFR* amplification, combined +7/−10) are available [2]. This cohort is therefore designated mixed-grade *IDH*-wildtype diffuse glioma, not glioblastoma, and is reported as a power-oriented sensitivity analysis rather than as an extension of the primary cohort (Supplementary Table S7).

### 2.10. Paired Primary-Recurrence Analysis

Within-patient change from initial diagnosis to first recurrence was tested with the two-sided Wilcoxon signed-rank test, and the effect size was estimated by the Hodges–Lehmann median of pairwise differences with its 95% confidence interval; the proportion of patients with increased expression is also reported. To test whether the observed change is attributable to a shift in specimen composition, tumour purity was compared between paired timepoints, the correlation between change in purity and change in *TMEM164* was computed, and linear models of the within-patient difference were fitted with an intercept alone, with change in purity, and with change in purity together with an ESTIMATE-derived score [26]. Tumour purity, ESTIMATE score, immune score and stromal score were taken as annotated in the GLASS clinical data distributed through cBioPortal; no scores were recomputed.

### 2.11. Protein-Level Assessment

To determine whether the transcript-level findings could be extended to protein, the CPTAC glioblastoma discovery cohort of 99 treatment-naive tumours [27] was interrogated through cBioPortal for *TMEM164* (Entrez Gene 84187), using *GAPDH*, *RIPK1*, *EGFR* and *SLC7A11* as positive controls, in both the protein-quantification and the z-scored matrices. The same query was extended to all seven CPTAC proteomic studies available through cBioPortal (Supplementary Table S9). Absence of a gene from a quantification matrix is reported as non-detection on that platform and is not interpreted as absence of the protein from tissue.

### 2.12. Statistical Software and Reproducibility

All analyses were performed in R version 4.6.1 (R Foundation for Statistical Computing, Vienna, Austria) using UCSCXenaTools (v1.7.0), survival (v3.8.11), rms (v8.1.1), Hmisc (v5.2.6), metafor (v5.0.1), metadat (v1.6.0), maxstat (v0.7.26), data.table (v1.18.6.1), dplyr (v1.2.1), reshape2 (v1.4.5), jsonlite (v2.0.0), curl (v8.0.0) and httr (v1.4.8); figures were produced with Python 3.10 (Python Software Foundation, Wilmington, DE, USA) and matplotlib 3.10. All tests were two-sided and *p* < 0.05 was considered nominally significant. A single seed (20260831) was used for every stochastic procedure. Analysis code, all derived analysis tables underlying the figures and tables, and the complete session information are deposited at Zenodo (doi: 10.5281/zenodo.22233721). This study is reported against the REMARK recommendations for prognostic biomarker studies [28]. An analysis of transcriptional cell-state signatures that was present in the originally submitted version has been removed: it contributed to none of the conclusions reported here, was not corrected for multiplicity, and was incompletely reported. It forms no part of the analyses described above.

## 3. Results

### 3.1. TMEM164 mRNA Is Higher in Glioblastoma than in Non-Tumour Brain

On the absolute expression scale, *TMEM164* was higher in tumour than in non-tumour brain in every comparison, but so were the constitutively expressed control transcripts. In CGGA-325, *TMEM164* rose by 1.287 log_2_ units (Cliff’s δ +0.767) while *ACTB* rose by 1.591 (δ +0.934) and *GAPDH* by 1.019 (δ + 0.867); in CGGA-693, the *TMEM164* difference did not reach significance (δ +0.216, *p* = 0.127) while *ACTB*, *GAPDH* and *TBP* all did. In TCGA, the pattern was less uniform, with *TBP* stable (*p* = 0.197) alongside significant shifts in *ACTB*, *GAPDH* and *TMEM164*. A global shift therefore affects these comparisons that library-size normalisation does not remove, and on the absolute scale a tumour-versus-normal difference cannot be attributed to biological overexpression rather than to technical or compositional difference (Supplementary Table S2).

**Figure 1:**
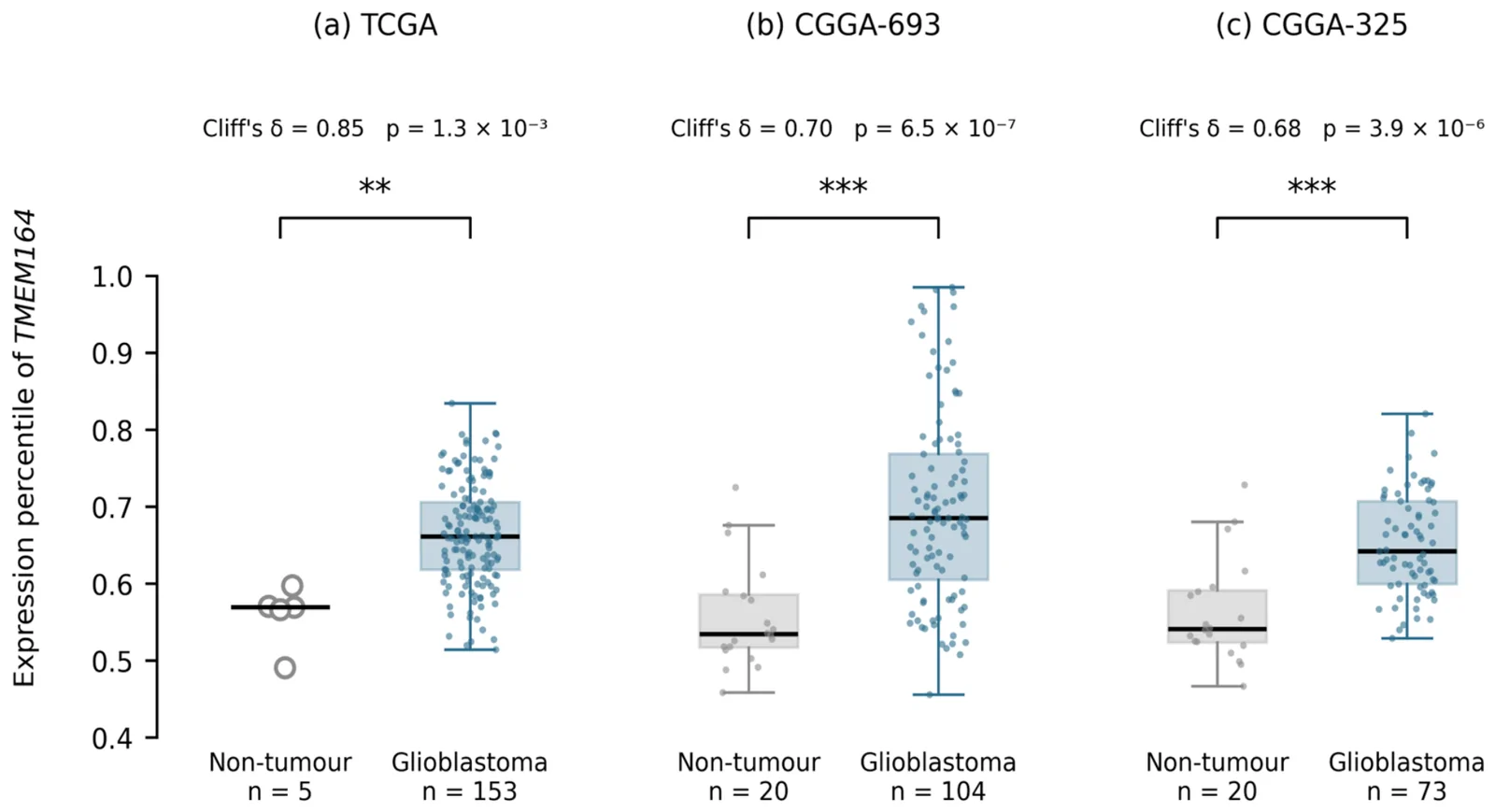
*TMEM164* expression relative to non-tumour brain, expressed as within-sample percentile rank among all measured transcripts. (**a**) TCGA; (**b**) CGGA-693; (**c**) CGGA-325. Boxes show median and interquartile range with individual samples overlaid; the five TCGA non-tumour samples are plotted individually with their median indicated. Groups were compared with the two-sided Wilcoxon rank-sum test and effect sizes are given as Cliff’s δ. ** *p* < 0.01, *** *p* < 0.001. The same 20 CGGA control samples serve panels (**b**) and (**c**), which are therefore not independent replications.

The within-sample percentile rank, which is invariant to global rescaling, resolves this. *TMEM164* occupied a higher rank among all transcripts in tumour than in non-tumour brain in all three cohorts: TCGA, median percentile 0.661 versus 0.570 (Cliff’s δ +0.848, 95% CI 0.723–0.948; *p* = 1.3 × 10^−3^); CGGA-693, 0.685 versus 0.535 (δ +0.705, 95% CI 0.515–0.862; *p* = 6.5 × 10^−7^); CGGA-325, 0.642 versus 0.541 (δ +0.677, 95% CI 0.437–0.881; *p* = 3.9 × 10^−6^) (Figure 1). In CGGA-693, where the absolute-scale comparison had collapsed, the percentile comparison was the strongest of the three, confirming that the limiting factor was scaling rather than signal. The percentile of the control samples was closely concordant between resources sequenced on different continents with different pipelines (0.535–0.570), which supports the stability of the measure itself.

Two controls confirm that this is not an artefact of the percentile scale. The per-sample fraction of detected genes did not differ between tumour and control samples in either CGGA comparison (medians 0.943 versus 0.932, *p* = 0.081; 0.930 versus 0.923, *p* = 0.102), and was marginally lower in TCGA tumours than in TCGA controls (0.861 versus 0.881, *p* = 3.3 × 10^−3^), a difference in the direction that would attenuate rather than inflate the observed tumour rank. Among the reference transcripts, *ACTB* and *GAPDH* occupy percentiles of 0.998 to 1.000 in both groups and shift by no more than 0.001, confirming that transcripts at the ceiling of the distribution cannot serve as controls on this scale; the ten uniformly expressed reference transcripts lie between 0.74 and 0.99 and shift by between −0.077 and +0.077, in both directions and without a systematic trend. *TBP*, the only control whose position in the distribution matches that of *TMEM164* (0.540, 0.614 and 0.620 in the three control sets), shifted by +0.010 (*p* = 0.609), +0.061 (*p* = 0.016) and +0.049 (*p* = 1.1 × 10^−4^), against +0.092, +0.151 and +0.101 for *TMEM164*. In the two CGGA cohorts the *TMEM164* shift therefore exceeds every reference transcript by a wide margin. In TCGA, it remains the largest in the panel but is approached by *CHMP2A* (+0.077), and the margin there is correspondingly narrow (Supplementary Table S11).

Two qualifications apply. Because the same 20 CGGA controls serve both CGGA cohorts, these are two independent tumour-versus-non-tumour comparisons, not three. And a higher within-sample rank in bulk tissue does not establish upregulation within any single cell type: tumour and normal cortex differ profoundly in cellular composition, and a shift in composition alone could produce this result. The five TCGA control values are plotted individually in Figure 1 rather than summarised, given their number.

### 3.2. TMEM164 Is Co-Expressed with RIPK1 Independently of Tumour Composition

**Figure 2:**
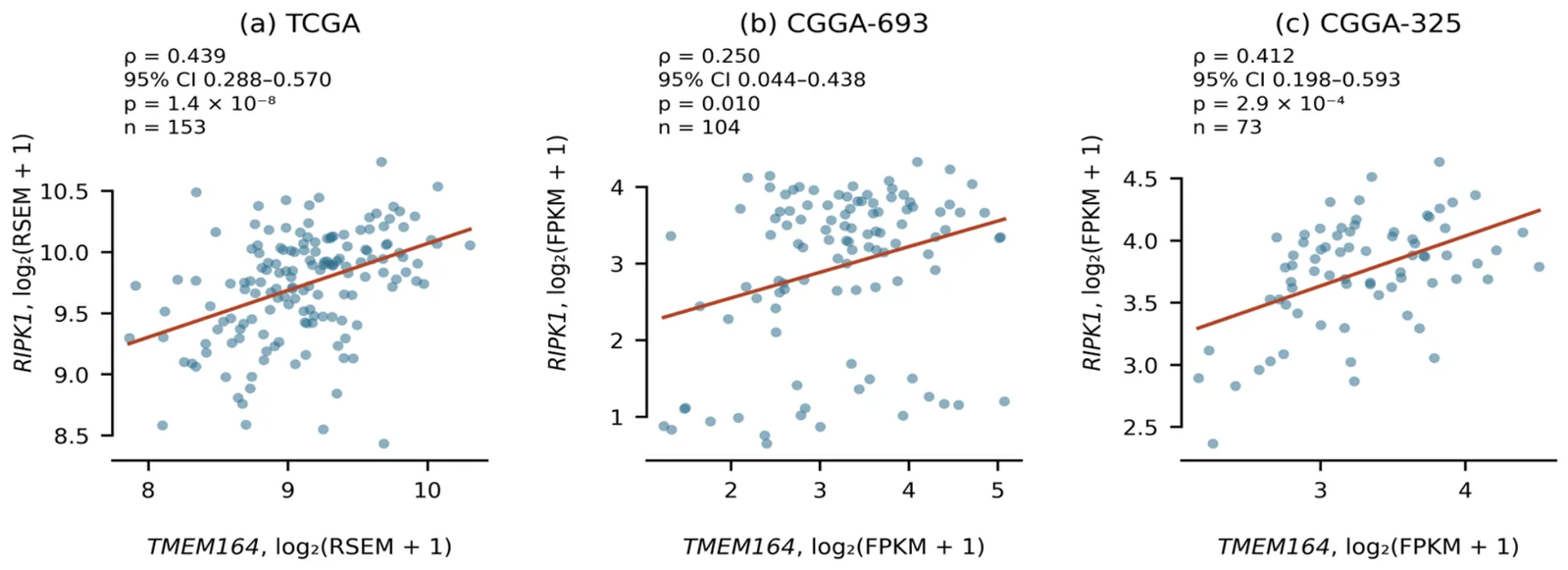
Co-expression of *TMEM164* and *RIPK1* in (**a**) TCGA, (**b**) CGGA-693 and (**c**) CGGA-325. Each panel reports the Spearman correlation with its percentile bootstrap 95% confidence interval (2000 resamples), the *p* value and the sample size. The regression line is shown for visualisation only; the reported statistic is the rank correlation.

*TMEM164* correlated positively with *RIPK1* in all three cohorts, and this was the only association among the four necroptosis- and lipid-related genes tested that survived correction for multiple comparisons in every cohort (Table 1, Figure 2). *RIPK3* and *MLKL* showed no consistent association. *TMEM164*-*FASN* correlation was nominally significant only in TCGA (ρ = 0.171, *p* = 0.035) and did not survive correction (q = 0.104), with no replication in either CGGA cohort. This analysis is descriptive: the mechanism proposed in the source study operates through direct protein interaction and enzymatic inhibition rather than transcription [8], and therefore predicts no particular transcript-level relationship in either direction.

**Figure 3:**
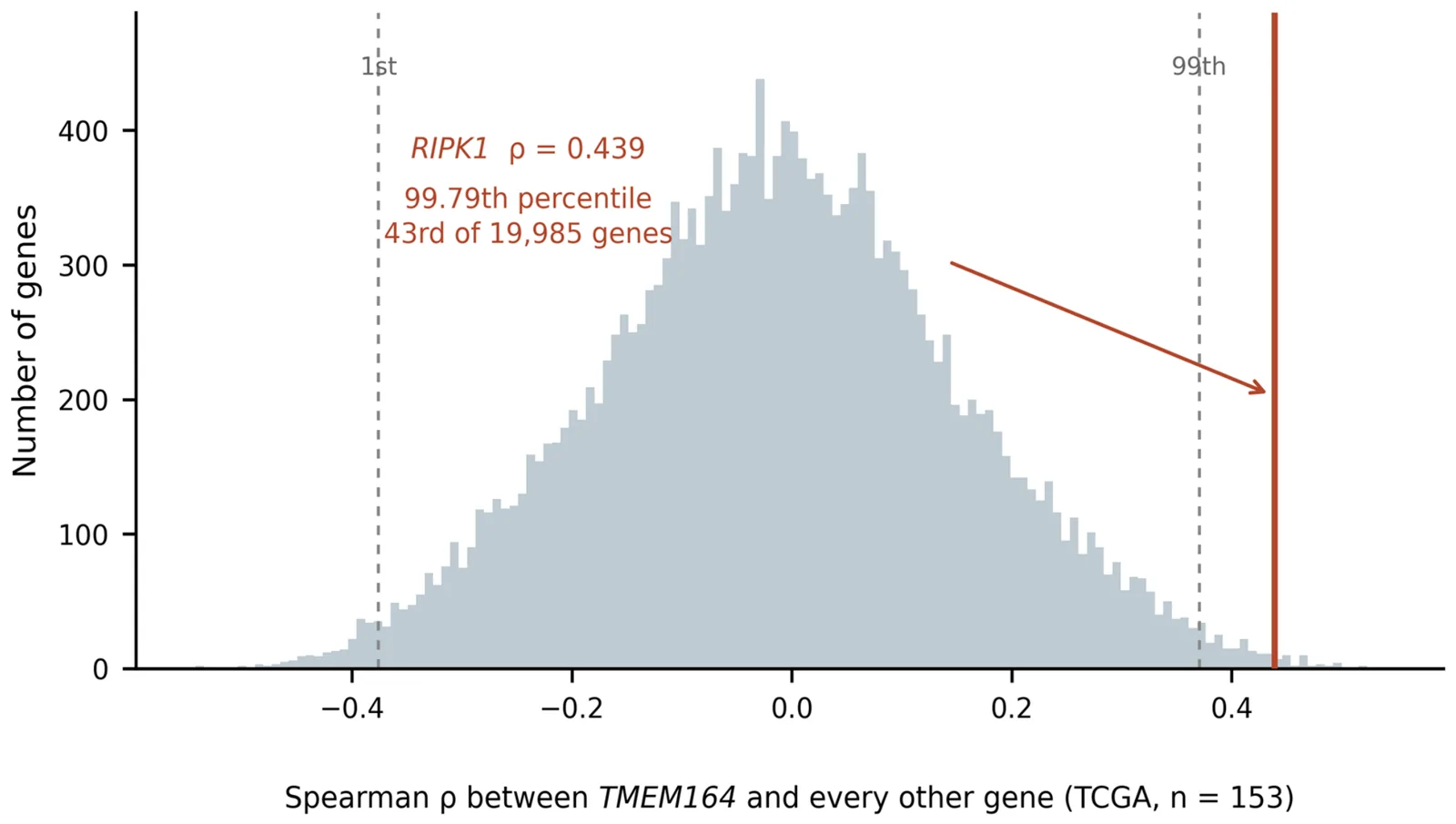
Transcriptome-wide null distribution of the Spearman correlation between *TMEM164* and each of 19,985 genes in TCGA. The red line marks the observed correlation with *RIPK1* (ρ = 0.439), which falls at the 99.79th percentile, with 42 genes correlating more strongly. Dashed lines mark the 1st and 99th percentiles of the null distribution.

Two analyses tested whether the *RIPK1* association reflects shared cellularity. Against a transcriptome-wide null of 19,985 genes in TCGA, ρ(*TMEM164*, *RIPK1*) = 0.439 fell at the 99.79th percentile: 42 genes correlated more strongly, placing *RIPK1* 43rd overall, against a null median of −0.011, a 1st-to-99th percentile range of −0.376 to 0.370, and a maximum of 0.539 (Figure 3). This was computed in TCGA, the cohort with the strongest observed correlation and therefore the condition least favourable to the null hypothesis. The three remaining genes tested fell within the body of the same null distribution, at the 86th (*FASN*), 82nd (*MLKL*) and 45th (*RIPK3*) percentiles (Supplementary Table S8), so the specificity of the *RIPK1* association is not shared by other necroptosis effectors, as a purely compositional explanation would predict. Among the 42 genes ranked above *RIPK1* were several innate-immune signalling transcripts (*TICAM1*, *NFKB1*, *STAT3*). This is reported descriptively, as no enrichment testing against an expression-matched background was performed; the transcriptome-wide correlation distribution is provided in the deposited analysis tables.

Partial Spearman correlation left the association essentially unchanged: 0.439 unadjusted, 0.437 adjusting for an immune/myeloid score, 0.440 for a stromal/endothelial score, and 0.437 for both. *TMEM164* itself correlated only weakly with the immune/myeloid score (ρ = 0.063), against 0.154 for *RIPK1*, indicating that *TMEM164* does not behave as a predominantly myeloid transcript in bulk glioblastoma. The co-expression is therefore not attributable to variation in immune, stromal or vascular content. It nonetheless remains a bulk-tissue observation and does not establish that the two transcripts are expressed in the same cells.

### 3.3. TMEM164 mRNA Shows No Replicable Association with Overall Survival

**Figure 4:**
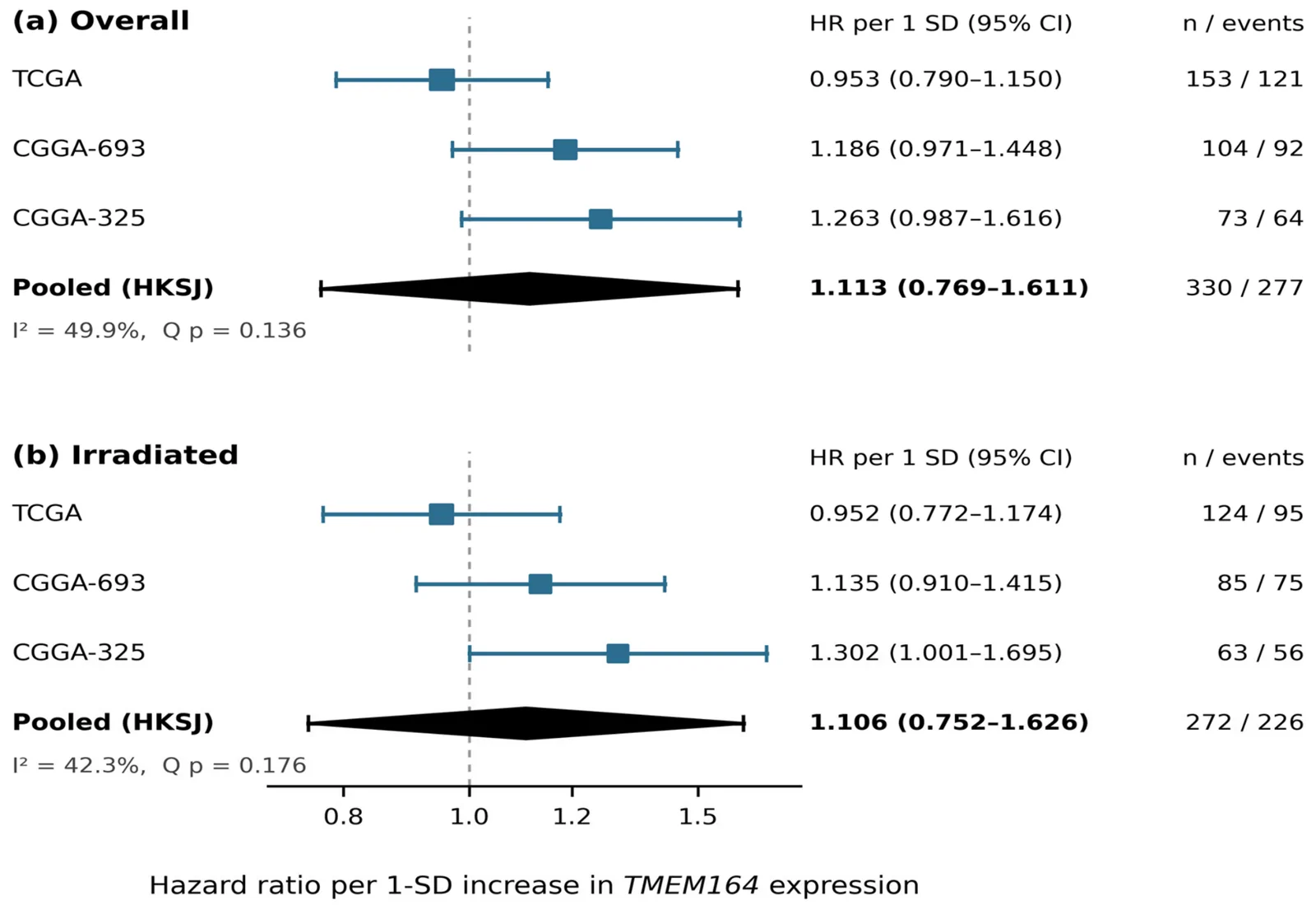
Association between continuous *TMEM164* expression and overall survival. (**a**) All patients; (**b**) patients with documented radiotherapy exposure. Squares indicate cohort-specific hazard ratios per one-standard-deviation increase, scaled by cohort size, with horizontal lines showing 95% confidence intervals; diamonds indicate the random-effects pooled estimate under the Hartung–Knapp–Sidik–Jonkman adjustment. The *x*-axis is logarithmic.

Continuous *TMEM164* expression was not significantly associated with overall survival in any cohort, and the point estimates pointed in opposite directions between resources: HR 0.953 per standard deviation in TCGA, 1.186 in CGGA-693 and 1.263 in CGGA-325 (Table 2, Figure 4). Under the Hartung–Knapp–Sidik–Jonkman adjustment used for primary inference, the pooled estimate was HR 1.113 (95% CI 0.769–1.611; *p* = 0.340), with τ^2^ = 0.0114, I^2^ = 49.9% and Cochran’s Q *p* = 0.136. Unadjusted REML gave the same point estimate with a narrower interval (1.113, 95% CI 0.938–1.320; *p* = 0.221) and a fixed-effect model gave 1.101 (95% CI 0.977–1.241; *p* = 0.115).

Two features of this synthesis deserve emphasis rather than reassurance. First, with only three cohorts, I^2^ is estimated imprecisely and Cochran’s Q has very little power, so a non-significant Q does not establish homogeneity; an I^2^ of approximately 50% is not low heterogeneity. Second, leave-one-out analysis showed that removing any single cohort destabilised the pooled interval severely: 1.216 (0.822–1.798) without TCGA, 1.084 (0.183–6.429) without CGGA-693, and 1.060 (0.265–4.238) without CGGA-325. The pooled estimate is therefore not a precise summary of a common effect, and the substantive finding is the failure of the cohort-level estimates to replicate one another in direction.

Model diagnostics supported the specification. The proportional-hazards assumption held in every cohort (global scaled Schoenfeld residual test *p* = 0.11, 0.43 and 0.24 for TCGA, CGGA-693 and CGGA-325). Refitting with a restricted cubic spline showed no departure from linearity (*p* = 0.33, 0.497, 0.608), so a threshold effect of the kind a dichotomised analysis would claim to capture is not present. Discrimination was close to chance: apparent C-index 0.453, 0.552 and 0.543, and after bootstrap correction for optimism 0.471, 0.544 and 0.536; the TCGA values lie below 0.5 because the hazard ratio in that cohort lies below unity. In every cohort, the 95% interval of the optimism estimate crossed zero, meaning the optimism was itself indistinguishable from zero, as expected for a predictor that does not discriminate.

In the TCGA primary multivariable model, adjusted for age and molecular *IDH* status and fitted on the full cohort of 153 patients with 121 events, *TMEM164* remained non-significant (HR 0.982, 95% CI 0.813–1.186; *p* = 0.849), while age was prognostic (HR 1.021 per year, 95% CI 1.005–1.039; *p* = 0.013). The secondary model substituting Karnofsky Performance Status was restricted to the 115 patients with complete KPS (89 events) and gave HR 0.963 (95% CI 0.768–1.206; *p* = 0.740).

These null results define a bounded exclusion rather than an absence of effect, and the boundary is set by the observed interval rather than by a power calculation. Under the Hartung–Knapp–Sidik–Jonkman model used for primary inference, the pooled interval extends to 1.61 per standard deviation, so hazard ratios above approximately 1.6 are not compatible with these data while smaller effects remain so; the unadjusted REML and fixed-effect models give the tighter upper bounds of 1.32 and 1.24. As a separate, design-stage quantity, the minimum hazard ratio per standard deviation that each cohort had 80% power to detect at a two-sided α of 0.05 was 1.29 in TCGA, 1.34 in CGGA-693 and 1.42 in CGGA-325, and 1.18 for the combined 277 events (Supplementary Table S4). These values describe the sensitivity of the design and are not themselves exclusion boundaries for the estimate obtained.

### 3.4. A Significant Prognostic Association Can Be Manufactured by Cutpoint Selection

Applying maximally selected rank statistics to the same expression values produced a nominally significant dichotomised association in two of three cohorts, and neither survived correction for the selection of the cutpoint (Table 3). In CGGA-693, the optimal threshold split the cohort into 54 high- and 50 low-expressing tumours and yielded HR 1.828 with a log-rank *p* of 0.0041, which rose to 0.094 after correction. In CGGA-325, a threshold placing 24 patients in the high group gave HR 1.792 with *p* 0.0295 uncorrected and 0.498 corrected. In TCGA, the optimal split ran in the opposite direction, with high expression apparently protective (HR 0.706), *p* 0.0825 uncorrected and 0.700 corrected.

This is a direct demonstration of how a positive dichotomised result arises in these data. The same values that show no association under continuous modelling produce a hazard ratio of 1.8 and a log-rank *p* below 0.005 once the threshold is chosen to maximise group separation, and the effect disappears once that selection is accounted for. That the procedure favours opposite directions in different cohorts, and that the optimal thresholds fall at different positions of the expression distribution (the 25th percentile in TCGA against roughly the median in CGGA-693 and the 67th in CGGA-325), further indicates that cutpoint-derived estimates here are not tracking a stable underlying quantity.

### 3.5. TMEM164 Is Not Associated with Survival Among Irradiated Patients

Because the mechanism under investigation concerns radioresistance, the survival analysis was repeated in patients with documented radiotherapy exposure. Radiotherapy status was available for 145 of 153 TCGA patients (95%) and for 96% of both CGGA cohorts, yielding an irradiated subgroup of 272 patients with 226 deaths.

No association replicated across cohorts. The cohort-level estimates were HR 0.952 (95% CI 0.772–1.174; *p* = 0.645) in TCGA, 1.135 (0.910–1.415; *p* = 0.261) in CGGA-693 and 1.302 (1.001–1.695; *p* = 0.0496) in CGGA-325, pooling to HR 1.106 under the Hartung–Knapp–Sidik–Jonkman model used for primary inference (95% CI 0.752–1.626; *p* = 0.379) and to 1.106 under unadjusted REML (0.928–1.317; *p* = 0.260), with I^2^ = 42.3% and Q *p* = 0.176 (Figure 4b). The CGGA-325 estimate is nominally significant at *p* = 0.0496—it is one of three cohort-level tests, is reproduced in neither of the other two, and is not treated here as evidence of an effect. Restricted to the two Chinese cohorts, which are the two whose point estimates already lie above unity, the same synthesis gives HR 1.201 (95% CI 1.014–1.423; *p* = 0.034) under a fixed-effect model, τ^2^ being estimated as zero, and HR 1.201 (0.509–2.835; *p* = 0.225) under the Hartung–Knapp adjustment. This is a two-cohort synthesis selected after inspection of the cohort estimates, and the corresponding estimate with TCGA removed from the full cohorts is HR 1.216 (95% CI 0.822–1.798; Supplementary Table S5). The divergence in direction between the North American and the Chinese resources seen in the full cohorts persists in the irradiated subgroup.

A *TMEM164* × radiotherapy interaction was tested in each cohort and did not replicate: *p* = 0.172 in TCGA, *p* = 0.0052 in CGGA-693 and *p* = 0.078 in CGGA-325. The CGGA-693 interaction was driven by 15 non-irradiated patients (HR 2.077 in that stratum), whereas the 21 non-irradiated TCGA patients showed no such pattern (HR 0.850, 95% CI 0.513–1.407; *p* = 0.528, 20 events). Patients who do not receive radiotherapy in a glioblastoma cohort differ systematically in performance status, extent of resection, age and comorbidity, so an interaction estimated within a non-irradiated stratum of this size is not interpretable as a treatment-modifying effect. Landmark analyses resetting the time origin at 60 and at 90 days in the CGGA cohorts did not materially alter the cohort-level estimates (Supplementary Table S6); this test has limited sensitivity here, because selection for fitness to receive radiotherapy operates before the landmark rather than within the landmark period.

Progression-free survival was not analysed. A progression-free interval is distributed for TCGA, but no comparably defined progression endpoint is annotated in either CGGA resource, and a single-cohort progression analysis would reintroduce precisely the single-database inference that this study sets out to test. The available progression endpoint is therefore reported as unanalysable rather than presented for one resource alone.

### 3.6. Sensitivity and Subgroup Analyses

None of the analyses in this section was planned in advance, none was corrected for multiplicity, and all are reported in full, including the results that do not support the study’s conclusion.

Molecularly confirmed *IDH*-wildtype TCGA cases. Restricting the analysis to the 143 patients with confirmed *IDH*-wildtype status (116 events) gave HR 0.970 per standard deviation (95% CI 0.803–1.172; *p* = 0.754) with adjustment for age, essentially identical to the full-cohort estimate. *TMEM164* expression did not differ between *IDH*-mutant and *IDH*-wildtype tumours (median 9.282 versus 9.126; *p* = 0.385). That the original histological selection had already excluded most *IDH*-mutant cases is evident from the fact that 25 *IDH1*-mutant tumours are present in the parent TCGA study against eight in this cohort.

*MGMT* promoter methylation. Four subgroup models were fitted. In CGGA-693, HR 1.031 (95% CI 0.772–1.378; *p* = 0.835) in methylated (*n* = 52, 46 events) and 1.089 (0.766–1.548; *p* = 0.635) in unmethylated tumours (*n* = 39, 36 events). In CGGA-325, HR 1.036 (0.761–1.411; *p* = 0.823) in unmethylated tumours (*n* = 48, 42 events), but HR 1.851 (95% CI 1.145–2.992; *p* = 0.012) in the 23 methylated tumours (20 events). This last result should not be accepted at face value. It rests on 23 patients, it was not planned in advance, it is one of four subgroup tests without multiplicity correction, and it is contradicted by the corresponding CGGA-693 subgroup, where the same stratum yields 1.031. It is precisely the class of finding that this study argues should not be treated as evidence, and reporting it under those qualifications is consistent with that argument rather than in tension with it.

Extended CGGA-693 cohort and tumour grade. CGGA-693 contains further primary, *IDH*-wildtype adult cases outside WHO grade IV, and analysing them addresses whether the null result in the grade IV cohort reflects limited power. Assembled irrespective of grade, this cohort comprises 164 patients with 130 events (26 WHO II, 34 WHO III, 104 WHO IV) and shows HR 1.228 (95% CI 1.043–1.446; *p* = 0.014), 1.194 (1.008–1.413; *p* = 0.040) after adjustment for grade, and 1.197 (1.013–1.415; *p* = 0.035) after adjustment for grade and age (Supplementary Table S7).

This association is not confounding by grade. The hazard ratio was almost identical within every stratum, at 1.186 (WHO II), 1.181 (WHO III) and 1.186 (WHO IV), the interaction between *TMEM164* and grade was null (likelihood-ratio *p* = 0.589), and grade itself remained strongly prognostic (WHO IV versus II, HR 3.459, 95% CI 1.878–6.372; *p* = 6.8 × 10^−5^). *TMEM164* was not monotonically related to grade: Spearman ρ 0.097 (*p* = 0.218), Kruskal–Wallis *p* = 0.037, with medians of 2.769, 3.400 and 3.304 across grades II, III and IV, so expression is lower only in WHO II and does not distinguish grade III from grade IV.

The correct interpretation is therefore not that *TMEM164* is prognostic in lower-grade tumours, but that CGGA-693 estimates the same weak positive effect of approximately 1.19 to 1.23 in every configuration, with 130 events instead of 92. This is a demonstration of the power argument within a single cohort. It does not reconcile with TCGA, which estimates 0.953 with 121 events. The discrepancy is between resources, not between grades, and it is what the pooled I^2^ of 49.9% measures. The exclusion of this cohort from the primary analysis is nonetheless justified on definitional rather than statistical grounds, because it cannot be classified under the 2021 WHO criteria with the annotation available in CGGA.

Cohort independence. CGGA-693 and CGGA-325 share no patients. GLASS contains 27 TCGA-format identifiers, of which nine of the 104 pairs (8.7%) include at least one TCGA sample and six patients also appear in the cross-sectional TCGA cohort; GLASS contains no CGGA cases. The three survival cohorts entering the meta-analysis are therefore mutually disjoint, whereas the longitudinal cohort partially overlaps with TCGA.

### 3.7. TMEM164 Expression Decreases Slightly at First Recurrence

**Figure 5:**
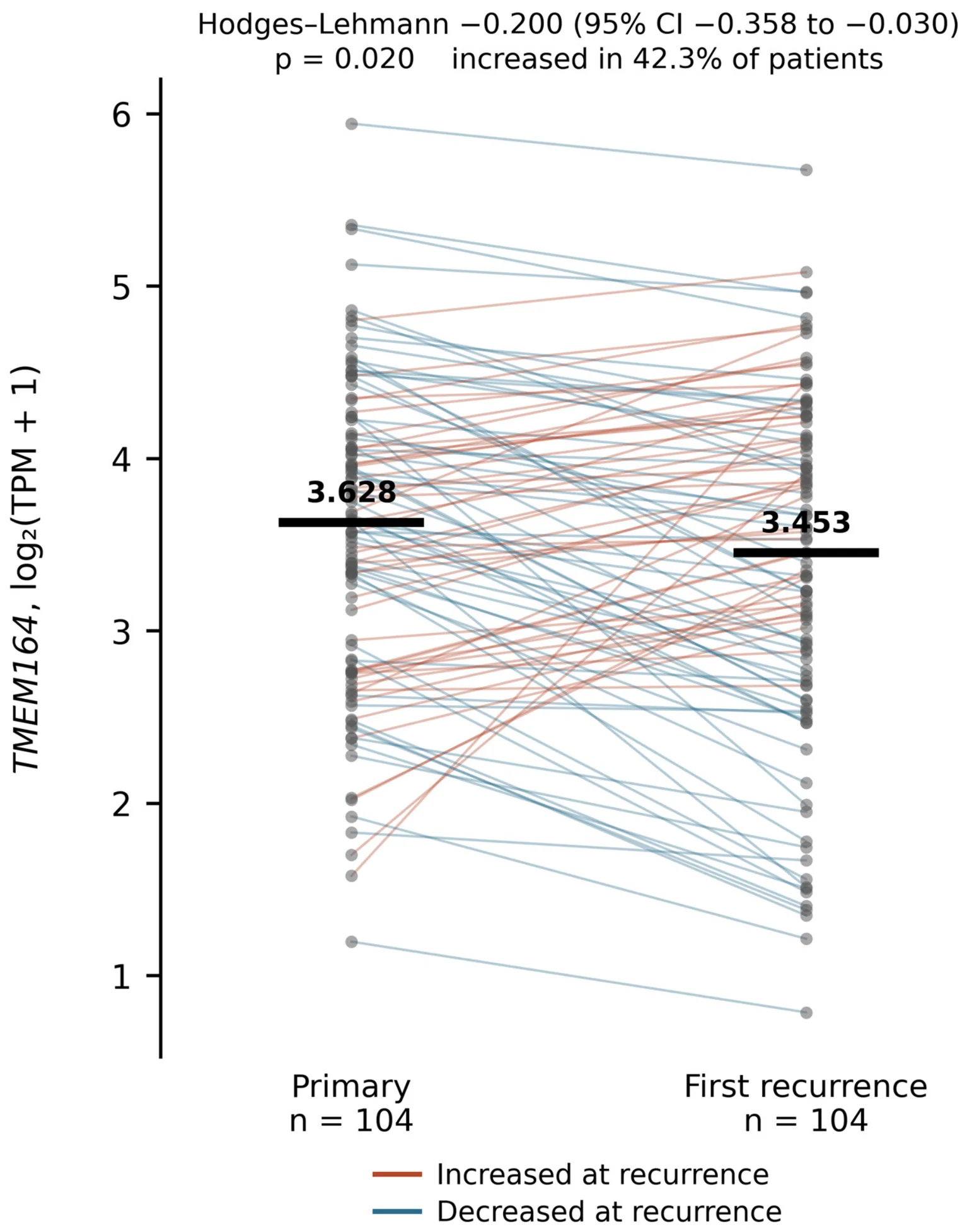
*TMEM164* expression in 104 matched primary and first-recurrence specimens from GLASS. Grey lines connect paired samples and are coloured by direction of change; black bars indicate group medians. The paired difference was tested with the two-sided Wilcoxon signed-rank test and quantified by the Hodges–Lehmann estimator.

Among the 104 matched GLASS pairs, median *TMEM164* expression fell from 3.628 to 3.453 log_2_(TPM + 1). The Hodges–Lehmann estimate of the paired difference was −0.200 log_2_ units (95% CI −0.358 to −0.030; *p* = 0.020), and expression increased in 42.3% of patients (Figure 5). On the ratio scale, this is a median decrease of 13%, with a 95% confidence interval spanning decreases of approximately 2% to 22%. The direction is opposite to the induction reported during fractionated irradiation in vitro [8].

The decrease is not explained by a shift in specimen composition. Tumour purity did not differ between paired timepoints (0.794 versus 0.792; *p* = 0.489) and change in purity was not correlated with change in *TMEM164* (ρ = 0.160; *p* = 0.104). Modelling the within-patient difference gave an intercept of −0.188 (95% CI −0.350 to −0.026; *p* = 0.023) unadjusted, −0.175 (−0.336 to −0.014; *p* = 0.033) adjusting for change in purity, and −0.196 (−0.362 to −0.030; *p* = 0.021) adjusting for change in purity together with the ESTIMATE score.

### 3.8. TMEM164 Protein Is Not Quantified in Public Glioblastoma Proteomes

Extension of these findings to the protein level was attempted using the CPTAC glioblastoma discovery cohort of 99 treatment-naive tumours [27]. TMEM164 protein was quantified in 0 of 99 samples, whereas *GAPDH*, *RIPK1*, *EGFR* and *SLC7A11* were each quantified in 99 of 99, and *TMEM164* mRNA was quantified in 99 of 99 within the same study. The result was identical in the z-scored matrix, excluding an error of gene identifier, sample list or query construction.

This non-detection is systematic rather than specific to glioblastoma. Across the seven CPTAC proteomic studies available through cBioPortal, *TMEM164* was detected in one (lung squamous carcinoma, 54 of 80 samples) while *RIPK1* was detected in six. In the lung adenocarcinoma proteome neither gene was detected (Supplementary Table S9). The pattern is consistent with the known under-representation of polytopic membrane proteins in tandem-mass-tag workflows and depends on the platform rather than on the tumour type.

An analysis of TMEM164 protein against overall survival, or against *RIPK1* phosphosites, is therefore not feasible with the currently accessible public proteomic resources. Absence from a quantification matrix constitutes non-detection on that platform and is not evidence that the protein is absent from tissue. Whether more recent proteomic or phosphoproteomic glioblastoma resources distributed outside cBioPortal would detect *TMEM164* could not be determined within the scope of this analysis.

## 4. Discussion

This multicohort reanalysis describes a consistent transcriptomic phenotype for *TMEM164* in adult glioblastoma and, at the same time, sets a boundary on what bulk transcript abundance can tell us about it. *TMEM164* mRNA occupied a higher within-sample rank in tumour than in non-tumour brain in all three cohorts, and was co-expressed with *RIPK1* in a manner that is highly specific against a transcriptome-wide background and unaffected by adjustment for immune, myeloid, stromal and endothelial content. Neither observation translated into reproducible prognostic information: no association with overall survival replicated across cohorts, in the pooled analysis, among irradiated patients, or among molecularly confirmed *IDH*-wildtype cases, and it decreased marginally rather than increased at first recurrence.

The tumour-versus-normal comparison required a change in method that is itself informative. On the absolute expression scale, constitutively expressed transcripts shifted between tumour and control as much as or more than *TMEM164* in the CGGA cohorts, so library-size normalisation alone was insufficient and no biological interpretation could be sustained. The within-sample percentile rank, which is invariant to global rescaling, recovered the difference consistently across resources sequenced on different continents with different pipelines. This qualifies but does not eliminate the interpretive limit: tumour tissue and normal cortex differ profoundly in cellular composition, and a higher rank in bulk tissue is compatible with a change in the mixture of cell types rather than upregulation within any one of them.

The reproducible co-expression with *RIPK1* is the study’s principal positive finding, and it is more specific than a bulk correlation would ordinarily be. Against 19,985 genes, *RIPK1* ranked 43rd in its correlation with *TMEM164*, partial correlation showed the association to be unmoved by composition, and the other three necroptosis- and lipid-related genes tested fell within the body of the same null distribution. It should nonetheless not be read as evidence of necroptotic activation. In the experimental model, the relevant endpoints are the phosphorylation of RIPK1, RIPK3 and MLKL, not their transcript abundance [8]; RIPK1 additionally has kinase-independent scaffolding functions supporting pro-survival NF-κB signalling [10,11]; and bulk co-expression cannot establish that two transcripts are present in the same cells.

The mechanism proposed in the source study is post-translational at both steps. TMEM164 was reported to interact with FASN and to inhibit its enzymatic activity, producing NADPH accumulation, neutralisation of radiation-induced reactive oxygen species, and suppression of necroptosis [8]. Such a model makes no prediction about the correlation between the two transcripts in either direction. Our *TMEM164*-*FASN* analysis is therefore descriptive and does not test that mechanism; the absence of a reproducible correlation is consistent with the mechanism rather than evidence against it. This distinction matters more broadly, because structural work indicates that TMEM164 acyltransferase output depends on dimerization and conformation and can vary non-linearly with protein abundance [7]. Redox balance and the choice between cell-death programmes are established as modifiable determinants of treatment response in glioblastoma, including through adjunct modalities that sensitise tumours to temozolomide by ROS-dependent mechanisms in vitro and in vivo [29], which is the level at which a TMEM164-directed intervention would have to be evaluated. The group that reported the TMEM164 mechanism has separately attributed radioresistance, in a glioblastoma model derived by repeated irradiation, to *IFI16*-driven and *HMOX1*-dependent suppression of ferroptosis [30], so the cell-death programme engaged after fractionated irradiation in these systems is itself context-dependent and is defined by protein activity rather than by steady-state transcription.

The absence of a prognostic association should be stated as a bounded exclusion rather than as an absence of effect, and the bound should be read from the observed interval rather than from a power calculation. Under the estimator used for primary inference the pooled interval extends to 1.61 per standard deviation, so effects above approximately 1.6 are not compatible with these data while smaller ones are; the cohorts were separately powered to detect 1.29, 1.34 and 1.42, which describes what the design could have seen rather than what it observed. Beyond power, the more instructive result is the failure of the cohort estimates to replicate: TCGA gives 0.953 with 121 events while both Chinese cohorts give estimates above unity, I^2^ is approximately 50%, and leave-one-out analysis destabilises the pooled interval so severely that the synthesis cannot be read as a precise summary of a common effect. That this divergence persists in the irradiated subgroup, and that the *TMEM164* × radiotherapy interaction did not replicate across cohorts, narrows the space in which a radiation-specific prognostic effect of baseline transcript abundance could still exist. Within CGGA-693, the same weak positive estimate of approximately 1.19 to 1.23 is obtained in every configuration, including a mixed-grade cohort with 130 events in which the effect is uniform across grades and does not interact with grade; the discrepancy is therefore between resources rather than between selection criteria.

The cutpoint analysis supplies the mechanism of the discrepancy with the source report. A hazard ratio of 1.8 with a log-rank *p* below 0.005 can be produced in these same data by choosing the threshold that maximises separation, and it does not survive correction for that choice, in either cohort where it occurs. This does not adjudicate the experimental findings of the source study, which concern protein activity in cell lines—it addresses only the supporting clinical analysis, and indicates that a dichotomised single-database Kaplan–Meier comparison with an unspecified threshold is not adequate evidence of prognostic relevance [9].

The small decrease at recurrence is unlikely to carry biological meaning. The confidence interval for the paired change spans decreases of approximately 2% to 22%, a range in which even the largest compatible change is of doubtful biological consequence; expression rose in 42.3% of patients, and the change was not attributable to a shift in tumour purity. GLASS specimens are separated by heterogeneous intervals and intervening therapies, whereas the reported induction of *TMEM164* occurs during fractionated irradiation. Recurrence-stage sampling therefore cannot exclude transient treatment-concurrent induction, and paired pre-treatment and on-treatment tissue would be required to test it.

Finally, the protein level, which is where the proposed mechanism resides, is currently inaccessible in public data. TMEM164 protein was quantified in none of the 99 CPTAC glioblastomas in which its mRNA was quantified in all 99, and it was detected in only one of seven CPTAC proteomes, a pattern consistent with the known under-representation of polytopic membrane proteins in tandem-mass-tag workflows. The implication is methodological rather than negative: testing this biology in patients will require targeted assays, immunohistochemistry with a validated antibody, targeted mass spectrometry, or phospho-specific measurement of necroptosis effectors, applied to tissue sampled in relation to treatment. Whether the montelukast S-enantiomer identified as a candidate TMEM164 inhibitor [7] can serve as a pharmacological tool in this setting will depend on central nervous system penetration and on selectivity data that are not yet available. Single-cell resolution of the expressing compartment is a required next step. *TMEM164* is expressed across brain cell lineages and most strongly in astrocytes, but it is downregulated in neurotoxic reactive astrocytes, in both patient single-nucleus data and cytokine-stimulated murine and human astrocytes, and astrocyte-specific overexpression preserves the homeostatic state [31]. A reactive astroglial contribution is therefore an unlikely source of the elevated bulk signal reported here, which strengthens rather than weakens the case that the signal arises in another compartment, although a bulk measurement cannot establish which.

### Limitations

Several limitations bound these conclusions. The comparison with non-tumour brain rests on five TCGA and 20 CGGA control samples, and because the same 20 CGGA controls serve both CGGA cohorts, it comprises two independent comparisons rather than three; bulk tissue cannot distinguish upregulation within a cell type from a change in cellular composition. The meta-analysis combines only three cohorts, so τ^2^ and I^2^ are estimated imprecisely and the pooled interval is unstable to the removal of any single cohort. The HKSJ adjustment was adopted for this reason but does not remove the underlying limitation. All subgroup, sensitivity and interaction analyses were exploratory, were not planned in advance, and were not corrected for multiplicity; the nominally significant hazard ratio in 23 *MGMT*-methylated CGGA-325 patients is reported for completeness and is contradicted by the corresponding CGGA-693 subgroup, as is the nominally significant estimate in the irradiated CGGA-325 subgroup, which is reproduced in neither of the other two cohorts. Molecular classification is not uniform across resources: *IDH* status was determined molecularly in all three survival cohorts, but the markers required to classify grade II and III *IDH*-wildtype astrocytic tumours as glioblastoma under the 2021 WHO classification are unavailable in CGGA. Six patients contribute to both the TCGA cohort and the GLASS pairs, so those two analyses are not fully independent, although the three cohorts entering the meta-analysis are mutually disjoint. Multivariable adjustment was limited by the covariates available in each resource. Above all, this is an analysis of steady-state transcript abundance, which cannot resolve cell-specific expression, protein abundance, subcellular localisation, enzymatic activity, post-translational modification, or causal function.

## 5. Conclusions

This study distinguishes the biological relevance of *TMEM164* from the prognostic utility of its baseline transcript abundance. The former remains an experimental question, whereas the latter is not supported reproducibly by current human glioblastoma datasets. Across independent adult glioblastoma cohorts, *TMEM164* mRNA occupies a consistently higher within-sample rank among all measured transcripts than in non-tumour brain and is co-expressed with *RIPK1* in a manner not explained by tumour composition, but it carries no reproducible prognostic information. The pooled interval excludes hazard ratios above approximately 1.6 per standard deviation, and the cohort-level estimates do not replicate one another in direction; a nominally significant association can be produced in the same data by selecting an optimal cutpoint and does not survive correction for that selection. Because TMEM164 protein is not quantified in the available public glioblastoma proteomes, the level at which the proposed radioresistance mechanism operates cannot currently be interrogated in patient material. Evaluation of *TMEM164* as a therapeutic target in glioblastoma should therefore proceed through protein- and activity-level assays on treatment-related tissue sampling, rather than through baseline bulk-tumour transcript abundance.

## Figures and Tables

**Table 1:** Co-expression of *TMEM164* with necroptosis effectors and *FASN*. Spearman ρ with percentile bootstrap 95% confidence interval (2000 resamples); q values from Benjamini–Hochberg correction across all 12 tests.

Cohort	Gene	*n*	ρ	95% CI	*p*	q
TCGA	*RIPK1*	153	0.439	0.288 to 0.570	1.4 × 10^−8^	1.7 × 10^−7^
TCGA	*RIPK3*	153	−0.031	−0.183 to 0.132	0.705	0.729
TCGA	*MLKL*	153	0.141	−0.013 to 0.287	0.081	0.195
TCGA	*FASN*	153	0.171	0.001 to 0.324	0.035	0.104
CGGA-693	*RIPK1*	104	0.250	0.044 to 0.438	0.010	0.041
CGGA-693	*RIPK3*	104	0.090	−0.124 to 0.286	0.361	0.592
CGGA-693	*MLKL*	104	0.078	−0.134 to 0.285	0.432	0.592
CGGA-693	*FASN*	104	0.063	−0.149 to 0.266	0.528	0.634
CGGA-325	*RIPK1*	73	0.412	0.198 to 0.593	2.9 × 10^−4^	1.8 × 10^−3^
CGGA-325	*RIPK3*	73	−0.041	−0.280 to 0.189	0.729	0.729
CGGA-325	*MLKL*	73	−0.091	−0.320 to 0.157	0.444	0.592
CGGA-325	*FASN*	73	−0.123	−0.339 to 0.097	0.300	0.592

**Table 2:** Association of *TMEM164* expression with overall survival by continuous Cox regression and random-effects meta-analysis.

Cohort	*n*	Events	HR per 1 SD	95% CI	*p*	C-Index
TCGA	153	121	0.953	0.790 to 1.150	0.616	0.471
CGGA-693	104	92	1.186	0.971 to 1.448	0.096	0.544
CGGA-325	73	64	1.263	0.987 to 1.616	0.064	0.536
Pooled, HKSJ (primary)	330	277	1.113	0.769 to 1.611	0.340	-
Pooled, REML	330	277	1.113	0.938 to 1.320	0.221	-
Pooled, fixed effect	330	277	1.101	0.977 to 1.241	0.115	-

τ^2^ = 0.0114; I^2^ = 49.9%; Cochran’s Q *p* = 0.136. C-index is the bootstrap optimism-corrected value and is reported directionally: a value below 0.5 indicates that higher expression is associated with longer survival, as in TCGA, where the hazard ratio lies below unity. HKSJ, Hartung–Knapp–Sidik–Jonkman; REML, restricted maximum likelihood; SD, standard deviation.

**Table 3:** Optimal cutpoint analysis by maximally selected rank statistics.

Cohort	Cutpoint	Group Sizes (High/Low)	HR (High vs. Low)	*p*, Log-Rank at Cutpoint	*p*, Corrected for Selection
TCGA	8.7945	114/39	0.706	0.0825	0.700
CGGA-693	3.2824	54/50	1.828	0.0041	0.094
CGGA-325	3.5534	24/49	1.792	0.0295	0.498

Cutpoints are on the native scale of each cohort, log_2_(RSEM + 1) for TCGA and log_2_(FPKM + 1) for CGGA. Correction for cutpoint selection follows Lausen and Schumacher [25].

## Data Availability

All primary data analysed in this study are publicly available: TCGA-GBM via the UCSC Xena platform (https://xenabrowser.net, accessed on 28 September 2026), CGGA mRNAseq_693, mRNAseq_325 and the non-glioma control dataset via http://www.cgga.org.cn, accessed on 28 September 2026 and GLASS and CPTAC via cBioPortal (https://www.cbioportal.org, accessed on 28 September 2026). The analysis code, all derived analysis tables underlying every figure and table, and the complete R session information are openly deposited at Zenodo (https://doi.org/10.5281/zenodo.22233721).

## Supplementary Materials

The following supporting information can be downloaded at: https://www.mdpi.com/article/10.3390/brainsci16101049/s1. Table S1: Cohort composition and availability of clinical covariates; Table S2: Tumour versus non-tumour comparison on the absolute expression scale, with constitutively expressed transcripts as negative controls; Table S3: Comparability of the CGGA non-tumour control dataset with the two CGGA tumour matrices; Table S4: Cox model diagnostics, discrimination and statistical power; Table S5: Meta-analytic sensitivity: alternative estimators and leave-one-out analysis; Table S6: Radiotherapy subgroup, interaction tests and landmark sensitivity analyses; Table S7: Extended CGGA-693 mixed-grade *IDH*-wildtype cohort (*n* = 164, 130 events); Table S8: Position of *TMEM164* co-expression partners within the transcriptome-wide null distribution (TCGA, 19,985 genes); Table S9: Detection of TMEM164 and RIPK1 protein across CPTAC proteomic studies in cBioPortal; Table S10: Subgroup analyses by *MGMT* promoter methylation and by molecular *IDH* status; Table S11: Controls on the within-sample percentile scale: per-sample detection fractions and percentile ranks of reference transcripts.
