# Supplementary material for: TMEM164 mRNA in Adult Glioblastoma: Multicohort Overexpression and RIPK1 Co-Expression Without Prognostic Replication
Source: Brain Sci. 2026 Sep 29;16(10):1049. doi: 10.3390/brainsci16101049 (PMC13702153; doi:10.3390/brainsci16101049)
Supplement: Supplementary file 1 [file brainsci-16-01049-s001.zip › brainsci-4502120-supplementary.pdf]

Supplementary Materials

TMEM164 mRNA in Adult Glioblastoma: Multicohort Overexpression and *RIPK1* Co-Expression Without Prognostic Replication

Table S1. Cohort composition and availability of clinical covariates.

Variable	TCGA	CGGA-693	CGGA-325	GLASS
Patients (n)	153	104	73	104 pairs
Deaths	121	92	64	not applicable
Expression unit	log_2(RSEM+1)	log_2(FPKM+1)	log_2(FPKM+1)	log_2(TPM+1)
Non-tumour controls	5	20 (shared)	20 (shared)	none
Tumour stage	primary only	primary only	primary only	paired primary / first recurrence
IDH status	molecular, 151/153	molecular, all	molecular, all	molecular, all
Age	available	available	available	available
Sex	available	available	available	available
Karnofsky score	115/153	not available	not available	not available
MGMT methylation	not available	91/104	71/73	not used
Radiotherapy status	145/153	available (96%)	available (96%)	not used
Chemotherapy status	not used	available	available	not used
WHO grade	not used	available	available	not used
1p/19q codeletion	not used	available	available	not used
Tumour purity	not used	not available	not available	104/104

Step	n
Samples in the TCGA-GBM expression matrix	172
Non-tumour brain samples (sample type code 11), used as within-platform reference	5
Recurrent tumour samples (code 02), all excluded	13
of which patients already represented by their own primary sample	6
of which patients represented only by a recurrent specimen	7
Primary tumour samples (code 01)	154
Excluded: absent from the curated survival matrix (TCGA-28-2510-01)	1
Final cohort, patients	153
Final cohort, deaths	121

TCGA case flow. The starting point is the TCGA-GBM expression matrix retrieved through UCSC Xena. Recurrent samples were excluded in full: six duplicated a patient already contributing a primary sample, with identical survival time and vital status, and seven were patients for whom no primary specimen is available. Treatment-naive status is documented rather than inferred from the sample type code: all 153 retained cases are annotated as primary tumour with no history of neoadjuvant treatment, although 5 carry a recorded history of prior glioma.

Covariates marked "not used" were present in the resource but not required by any model reported here; those marked "not available" were not annotated in the distributed clinical table for that resource, which is why *MGMT* promoter methylation could not enter the TCGA models. The 20 CGGA control samples are the same for both CGGA cohorts.

Table S2. Tumour versus non-tumour comparison on the absolute expression scale, with constitutively expressed transcripts as negative controls.

Cohort	Gene	Controls	Tumours	Δ log_2	Cliff's δ (95% CI)	p
CGGA-693	*TMEM164*	2.468	3.117	+0.650	+0.216 (−0.010 to +0.425)	0.127
CGGA-693	*RIPK1*	2.663	3.393	+0.731	+0.368 (+0.174 to +0.560)	0.0094
CGGA-693	*ACTB*	10.665	11.967	+1.302	+0.498 (+0.329 to +0.653)	4.4×10^{-4}
CGGA-693	*GAPDH*	11.056	11.752	+0.696	+0.407 (+0.228 to +0.572)	0.0041
CGGA-693	*TBP*	3.237	3.635	+0.398	+0.340 (+0.151 to +0.521)	0.0163

CGGA-325	*TMEM164*	2.468	3.755	+1.287	+0.767 (+0.559 to +0.937)	1.7×10^{-7}
CGGA-325	*RIPK1*	2.663	4.387	+1.724	+0.951 (+0.878 to +0.996)	8.9×10^{-11}
CGGA-325	*ACTB*	10.665	12.256	+1.591	+0.934 (+0.863 to +0.988)	1.9×10^{-10}
CGGA-325	*GAPDH*	11.057	12.075	+1.019	+0.867 (+0.752 to +0.953)	3.3×10^{-9}
CGGA-325	*TBP*	3.237	3.953	+0.716	+0.881 (+0.778 to +0.962)	1.9×10^{-9}
TCGA	*TMEM164*	8.316	9.126	+0.811	+0.890 (+0.796 to +0.963)	7.3×10^{-4}
TCGA	*ACTB*	15.939	16.986	+1.047	–	3.2×10^{-4}
TCGA	*GAPDH*	15.817	16.428	+0.611	–	0.0059
TCGA	*TBP*	8.101	8.294	+0.193	–	0.197

CGGA values are $\log_2$(CPM + 1) recomputed over the common gene space; TCGA values are $\log_2$(RSEM + 1) as distributed. Group values are medians. The constitutively expressed controls shift as much as or more than *TMEM164* in the CGGA cohorts, so a global scale difference is present that library-size normalisation does not remove. The five individual TCGA non-tumour values were 7.479, 8.293, 8.316, 8.365 and 8.534 against a tumour range of 7.864 to 10.303. Effect sizes are not reported for the TCGA housekeeping transcripts because with five control samples the estimator of the variance of Cliff's δ is unstable; those comparisons are given on p values alone, and the TCGA intervals throughout this table should be read as indicative rather than as precise bounds.

Table S3. Comparability of the CGGA non-tumour control dataset with the two CGGA tumour matrices.

Quantity	Control set	CGGA-693	CGGA-325
Annotated genes	55,523	23,987	24,326
Genes shared with control set	–	23,987 (100%)	24,326 (100%)
Median library fraction retained within the common gene space	0.999	0.999	0.999

Counts per million were recomputed separately for each cohort over the corresponding common gene space, so control values were derived independently for the two comparisons; the resulting numerical difference between them is negligible at the precision reported in Table S2.

Table S4. Cox model diagnostics, discrimination and statistical power.

Cohort	Schoenfeld p (global)	Spline non-linearity p	C-index, apparent	C-index, optimism-corrected (95% CI of optimism)
TCGA	0.11	0.33	0.453	0.471 (−0.029 to +0.070)
CGGA-693	0.43	0.497	0.552	0.544 (−0.054 to +0.076)
CGGA-325	0.24	0.608	0.543	0.536 (−0.069 to +0.093)

Cohort	Events	Minimum HR per SD detectable at 80% power
TCGA	121	1.29
CGGA-693	92	1.34
CGGA-325	64	1.42
Pooled	277	1.18

Optimism corrected by bootstrap with 2000 resamples (seed 20260831). The concordance index is reported directionally, so that a value below 0.5 denotes discrimination in the direction opposite to the modelled one. This is the case in TCGA, where the point estimate for TMEM164 lies below unity and both the apparent and the optimism-corrected values therefore fall below 0.5; optimism correction moves the estimate towards 0.5 in that cohort, which is the expected behaviour on the directional scale. The minimum detectable hazard ratios are design-stage quantities derived from the observed number of events at 80% power and a two-sided alpha of 0.05, the pooled row using the combined 277 events. They describe the sensitivity conferred by the sample sizes and are not the exclusion boundary for the observed synthesis, which is read from the confidence interval of the pooled estimate in Table S5.

Table S5. Meta-analytic sensitivity: alternative estimators and leave-one-out analysis.

Model	Pooled HR	95% CI	p
Random effects, HKSJ (primary)	1.113	0.769 to 1.611	0.340
Random effects, REML	1.113	0.938 to 1.320	0.221
Fixed effect	1.101	0.977 to 1.241	0.115
Leave-one-out: without TCGA	1.216	0.822 to 1.798	–
Leave-one-out: without CGGA-693	1.084	0.183 to 6.429	–

Leave-one-out: without CGGA-325	1.060	0.265 to 4.238	–

τ^2 = 0.0114; I^2 = 49.9%; Cochran's Q p = 0.136. With only three cohorts Cochran's Q has very little power, so a non-significant Q does not establish homogeneity. Leave-one-out intervals use the HKSJ adjustment on two cohorts and are shown to illustrate instability, not for inference. The upper bound of the primary HKSJ interval, 1.61 per standard deviation, is the exclusion boundary supported by these data.

Table S6. Radiotherapy subgroup, interaction tests and landmark sensitivity analyses.

Cohort	n	Events	HR per SD	95% CI	p
TCGA, irradiated	124	95	0.952	0.772 to 1.174	0.645
CGGA-693, irradiated	85	75	1.135	0.910 to 1.415	0.261
CGGA-325, irradiated	63	56	1.302	1.001 to 1.695	0.0496
Pooled, HKSJ	272	226	1.106	0.752 to 1.626	0.379
Pooled, REML	272	226	1.106	0.928 to 1.317	0.260
TCGA, not irradiated	21	20	0.850	0.513 to 1.407	0.528

Test	p
TMEM164 × radiotherapy interaction, TCGA	0.172
TMEM164 × radiotherapy interaction, CGGA-693	0.0052
TMEM164 × radiotherapy interaction, CGGA-325	0.078

I^2 = 42.3%; Q p = 0.176. Landmark analyses resetting the time origin at 60 and at 90 days were performed in the CGGA cohorts only and did not materially alter the cohort-level estimates. Pooling the two irradiated CGGA subgroups alone gives HR 1.201 (95% CI 1.014 to 1.423; p = 0.034) under a fixed-effect model, τ^2 being estimated as zero, and HR 1.201 (0.509 to 2.835; p = 0.225) under the Hartung-Knapp adjustment. This is not interpreted as evidence of a prognostic effect: it is a two-cohort synthesis restricted to the two resources whose estimates already lie above unity, and the corresponding full-cohort estimate with TCGA removed is HR 1.216 (95% CI 0.822 to 1.798; Table S5). The landmark test has limited sensitivity here because selection for fitness to receive radiotherapy operates before the landmark rather than within the landmark period.

Table S7. Extended CGGA-693 mixed-grade *IDH*-wildtype cohort (n = 164, 130 events).

Model / stratum	n	HR per SD	95% CI	p
TMEM164 alone	164	1.228	1.043 to 1.446	0.014
+ WHO grade	164	1.194	1.008 to 1.413	0.040
+ WHO grade + age	164	1.197	1.013 to 1.415	0.035
WHO grade II only	26	1.186	0.684 to 2.056	0.544
WHO grade III only	34	1.181	0.800 to 1.744	0.403
WHO grade IV only	104	1.186	0.971 to 1.448	0.096
WHO grade II + III	60	1.292	0.967 to 1.726	0.083

Additional test	Result
WHO IV vs II (adjusted for *TMEM164*)	HR 3.459 (1.878 to 6.372), p = 6.8×10^{-5}
TMEM164 × grade interaction (likelihood ratio)	p = 0.589
TMEM164 vs grade, Spearman	ϱ = 0.097, p = 0.218
TMEM164 across grades, Kruskal–Wallis	p = 0.037

Grade-specific medians: 2.769 (II), 3.400 (III), 3.304 (IV) $\log_2$(FPKM+1). This cohort cannot be classified under the 2021 WHO criteria because *TERT* promoter mutation, *EGFR* amplification and +7/−10 status are not annotated in CGGA. Event counts by stratum are 12 (WHO II), 26 (WHO III), 92 (WHO IV) and 38 (WHO II and III combined), against 130 in the full extended cohort.

Table S8. Position of *TMEM164* co-expression partners within the transcriptome-wide null distribution (TCGA, 19,985 genes).

Gene	Spearman ϱ	Percentile in null	Genes correlating more strongly	Rank
RIPK1	+0.439	99.79	42	43
FASN	+0.171	86.39	2,720	2,721
MLKL	+0.141	82.45	3,508	3,509

RIPK3	−0.031	45.03	10,986	10,987

Null distribution statistic	Value
Median	−0.011
1st percentile	−0.376
5th percentile	−0.282
95th percentile	+0.268
99th percentile	+0.370
Minimum	−0.543
Maximum	+0.539

Computed in TCGA, the cohort with the strongest observed *TMEM164*–*RIPK1* correlation and therefore the condition least favourable to the null hypothesis. Only *RIPK1* lies outside the bulk of the distribution.

Table S9. Detection of *TMEM164* and *RIPK1* protein across CPTAC proteomic studies in cBioPortal.

CPTAC study	*RIPK1*, samples quantified	*TMEM164*, samples quantified
Glioblastoma (gbm_cptac_2021)	99	0
Brain, paediatric (brain_cptac_2020)	218	0
Breast (brca_cptac_2020)	122	0
Pancreas (paad_cptac_2021)	140	0
Endometrium (ucec_cptac_2020)	81	0
Lung adenocarcinoma (luad_cptac_2020)	0	0
Lung squamous (lusc_cptac_2021)	80	54

In gbm_cptac_2021, *TMEM164* mRNA was quantified in 99 of 99 samples and *GAPDH*, *EGFR* and *SLC7A11* protein in 99 of 99, excluding an error of identifier or query. Results were identical in the z-scored matrices. Absence from a quantification matrix indicates non-detection on that platform, not absence of the protein from tissue.

Table S10. Subgroup analyses by *MGMT* promoter methylation and by molecular *IDH* status.

Subgroup	n	Events	HR per SD	95% CI	p
CGGA-693, *MGMT* methylated	52	46	1.031	0.772 to 1.378	0.835
CGGA-693, *MGMT* unmethylated	39	36	1.089	0.766 to 1.548	0.635
CGGA-325, *MGMT* methylated	23	20	1.851	1.145 to 2.992	0.012
CGGA-325, *MGMT* unmethylated	48	42	1.036	0.761 to 1.411	0.823
TCGA, *IDH*-wildtype confirmed	143	116	0.970	0.803 to 1.172	0.754
TCGA, *IDH* profiled only	151	119	0.981	0.813 to 1.185	0.845

TCGA models adjusted for age. None of these analyses was planned in advance and none was corrected for multiplicity. The CGGA-325 methylated subgroup comprises 23 patients, was one of four subgroup tests, and is contradicted by the corresponding CGGA-693 subgroup; it should not be interpreted as evidence of a prognostic effect. *TMEM164* expression did not differ between *IDH*-mutant and *IDH*-wildtype tumours (median 9.282 vs 9.126; p = 0.385).

Table S11. Controls on the within-sample percentile scale: per-sample detection fractions and percentile ranks of reference transcripts.

(a) Per-sample fraction of genes detected.

Cohort	Genes analysed	Tumour, median (IQR)	Non-tumour, median (IQR)	p
TCGA	20,530	0.861 (0.849–0.868)	0.881 (0.880–0.881)	3.3×10^{-3}
CGGA-693	23,987	0.943 (0.916–0.955)	0.932 (0.919–0.940)	0.081
CGGA-325	24,326	0.930 (0.911–0.945)	0.923 (0.911–0.930)	0.102

(b) Within-sample percentile rank of *TMEM164* and of the reference transcripts.

**Cohort**	**Transcript**	**Non-tumour, median percentile**	**Tumour, median percentile**	**Shift**	**p**
TCGA	*TMEM164*	0.570	0.661	+0.092	1.3×10^{-3}
TCGA	*TBP*	0.540	0.551	+0.010	0.609
TCGA	*C1orf43*	0.939	0.971	+0.032	2.9×10^{-3}
TCGA	*CHMP2A*	0.848	0.925	+0.077	1.9×10^{-3}
TCGA	*EMC7*	not annotated	–	–	–
TCGA	*GPI*	0.992	0.978	−0.014	0.011
TCGA	*PSMB4*	0.921	0.971	+0.049	1.6×10^{-4}
TCGA	*RAB7A*	0.974	0.984	+0.010	6.6×10^{-4}
TCGA	*REEP5*	0.987	0.959	−0.027	5.1×10^{-4}
TCGA	*SNRPD3*	0.738	0.806	+0.068	6.7×10^{-3}
TCGA	*VCP*	0.975	0.979	+0.004	0.481
TCGA	*VPS29*	0.778	0.822	+0.044	0.025
TCGA	*ACTB*	1.000	1.000	0.000	7.9×10^{-3}
TCGA	*GAPDH*	1.000	1.000	0.000	0.559
CGGA-693	*TMEM164*	0.535	0.686	+0.151	6.5×10^{-7}
CGGA-693	*TBP*	0.614	0.675	+0.061	0.016
CGGA-693	*C1orf43*	0.977	0.941	−0.037	1.8×10^{-12}
CGGA-693	*CHMP2A*	0.973	0.979	+0.006	0.071
CGGA-693	*EMC7*	0.945	0.928	−0.017	1.1×10^{-5}
CGGA-693	*GPI*	0.968	0.972	+0.004	0.135
CGGA-693	*PSMB4*	0.980	0.986	+0.006	1.6×10^{-3}
CGGA-693	*RAB7A*	0.976	0.958	−0.018	6.3×10^{-7}
CGGA-693	*REEP5*	0.957	0.880	−0.077	5.0×10^{-10}
CGGA-693	*SNRPD3*	0.917	0.913	−0.004	0.589
CGGA-693	*VCP*	0.924	0.928	+0.004	0.783
CGGA-693	*VPS29*	0.952	0.928	−0.025	3.0×10^{-7}
CGGA-693	*ACTB*	0.998	0.999	+0.001	1.6×10^{-3}
CGGA-693	*GAPDH*	0.999	0.999	0.000	7.4×10^{-3}
CGGA-325	*TMEM164*	0.541	0.642	+0.101	3.9×10^{-6}
CGGA-325	*TBP*	0.620	0.669	+0.049	1.1×10^{-4}
CGGA-325	*C1orf43*	0.978	0.971	−0.006	8.3×10^{-4}
CGGA-325	*CHMP2A*	0.973	0.977	+0.004	0.100
CGGA-325	*EMC7*	0.946	0.939	−0.007	4.8×10^{-3}
CGGA-325	*GPI*	0.968	0.976	+0.008	0.050
CGGA-325	*PSMB4*	0.980	0.985	+0.005	3.2×10^{-5}
CGGA-325	*RAB7A*	0.976	0.983	+0.007	3.4×10^{-7}
CGGA-325	*REEP5*	0.957	0.927	−0.031	6.3×10^{-6}
CGGA-325	*SNRPD3*	0.918	0.942	+0.024	4.0×10^{-4}
CGGA-325	*VCP*	0.925	0.960	+0.035	7.1×10^{-9}
CGGA-325	*VPS29*	0.953	0.942	−0.011	4.2×10^{-4}
CGGA-325	*ACTB*	0.998	0.999	+0.001	3.0×10^{-10}
CGGA-325	*GAPDH*	0.999	0.999	+0.001	1.1×10^{-6}

Percentile ranks were computed within each sample over the gene space common to the tumour and control matrices of the corresponding comparison, which is the same space used for *TMEM164* in Figure 1. Group values are medians; the shift is the difference between the tumour and the non-tumour median. Groups were compared with the two-sided Wilcoxon rank-sum test and no multiplicity correction was applied, these comparisons being controls rather than hypothesis tests. The ten reference transcripts are drawn from the set of uniformly expressed genes proposed for calibration by Eisenberg and Levanon (Eisenberg E, Levanon EY. Human housekeeping genes, revisited. *Trends Genet.* 2013;29:569–574; doi:10.1016/j.tig.2013.05.010); *EMC7* is not annotated in the TCGA expression matrix and could not be evaluated in that cohort. *ACTB* and *GAPDH* occupy the top of the distribution in every sample and are retained to show that transcripts at the ceiling cannot serve as controls on this

scale. *TBP* is the only reference transcript whose position in the control distribution is comparable to that of *TMEM164* and therefore the only one with equivalent room to move in either direction.
